# Metabolomic and Transcriptomic Analyses of Flavonoid Biosynthesis in *Dendrobium devonianum* Flowers

**DOI:** 10.3390/genes16030264

**Published:** 2025-02-24

**Authors:** Yue Li, Yawen Wu, Ran Pu, Xuejiao Li, Tian Bai, Nengbo Li, Ying Zhou, Jingli Zhang

**Affiliations:** 1 College of Horticulture and Landscape, Yunnan Agricultural University, Kunming 650201, China; liyue12030625@163.com (Y.L.); yawenwu@ynau.edu.cn (Y.W.); puran1026@163.com (R.P.); lixuejiao@ynau.edu.cn (X.L.); tbai@ynau.edu.cn (T.B.); 2 Institute of Caulis Dendrobii of Longling County, Longling 678300, China; lnb0805@126.com

**Keywords:** *Dendrobium devonianum*, transcriptome, metabolome, flavonoids

## Abstract

Background: *Dendrobium devonianum* is a traditional Chinese medicinal herb with notable ornamental and medicinal value. Methods: In this study, transcriptomic and metabolomic approaches were employed to investigate gene expression and secondary metabolite changes during four developmental stages of *D. devonianum* flowers. Results: Metabolomic analysis identified 1186 distinct metabolites, with flavonoid compounds being the most abundant category (213 types). Transcriptomic analysis revealed 31 differentially expressed genes associated with flavonoid biosynthesis and flavonoid and flavonol biosynthesis pathways. Among these, key genes regulating flavonol synthesis, including *F3H* (Unigene0077194) and *FLS* (Unigene0062137), exhibited high expression levels in the early developmental stage (S1). Conclusions: Flavonoids serve as the major active components in *D. devonianum* flowers, exhibiting a wide range of pharmacological properties. This study provides valuable insights into the molecular mechanisms driving flavonoid accumulation in *D. devonianum*, offering a foundation for further functional studies and applications in ornamental and medicinal plant research.

## 1. Introduction

Dendrobium, the largest genus in the Orchidaceae family, includes approximately 1500 species distributed across Asia, Europe, Oceania, and other regions [1]. In China, over 80 species of Dendrobium have been reported [2]. Many species within this genus are highly prized in traditional medicine, particularly for their stems, commonly used in cases of heat-induced damage to body fluids, stomach pain with dry retching, and dry cough due to lung dryness [3]. *D. devonianum* is a perennial epiphytic herb [4]. In China, it is regarded as an important medicinal and edible plant [5], valued both for its medicinal stems and ornamental appeal. The chemical composition of *D. devonianum* is diverse, with reported constituents including alkaloids, polysaccharides, flavonoids, phenolic acids, steroids, fatty acids, as well as amino acids, trace elements, and other compounds [6,7]. With the increasing value of Dendrobium stems, large quantities of flowers have been produced during intensive cultivation, drawing the attention of researchers. Although the flowers are not the primary harvested material, recent studies have highlighted their rich content of nutrients and bioactive compounds, including antioxidant, hepatoprotective, hypoglycemic, and antihypertensive agents [8]. In traditional practices, people often consume *D. devonianum* flowers by infusing them in water, wine, or juice, or by boiling them in porridge or soup, believing they help nourish the yin, moisturize the lungs, beautify the skin, calm the mind, relieve stress, and promote stomach health [9]. Modern research has revealed that *D. devonianum* flowers contain polysaccharides, polyphenols, various amino acids, crude proteins, flavonoids, essential minerals, and volatile compounds [10,11]. Studies have also confirmed that ethanol extracts, polysaccharides, and amino acids from the flowers exhibit significant in vitro antioxidant properties [12,13].

In recent years, flavonoid compounds in Dendrobium have attracted significant attention for their potent antioxidant activities and remarkable capacity to scavenge free radicals [14,15]. Liao Xian et al. [16] isolated five flavonoid compounds from the stems of *D. devonianum*, including vitexin II, vitexin I, Schaftoside, isoschaftoside, and rutin. Shen Yan et al. [17] identified one dihydroflavonoid and four flavonoid glycosides from the stems of *D. devonianum*. Zhao Ming et al. [18] found six flavonoid compounds in *D. devonianum* flowers, including taxifolin, luteolin, and quercetin. Flavonoids are widespread secondary metabolites in plants that play a crucial role in plant growth and development and help plants to resist biotic and abiotic stresses. [19]. They exhibit a range of pharmacological effects, including antioxidant, free radical scavenging, anticancer, cholesterol-lowering, and antimicrobial properties [20], thus holding great potential for pharmaceutical development and therapeutic applications. Current research on *D. devonianum* mainly focuses on the bioactive components, pharmacological effects, and toxicology of the stems, while studies on the flavonoid compounds in the flowers are relatively scarce.

Studies have revealed that the molecular mechanisms in plants are characterized by the involvement of multiple targets, pathways, and interconnected network processes [21]. To explore the biosynthetic pathways of diverse metabolites in plants, multiple technologies, including transcriptomics, proteomics, and metabolomics, have been integrated into plant research. These approaches have successfully been used to identify key genes and metabolites in various plants, including *Dendrobium officinale*, Chinese sour jujube, and Gardenia [22,23,24], thereby expanding the development of biosynthesis pathways for bioactive compounds in plants. Shen et al. investigated the biosynthesis mechanisms of polysaccharides and alkaloids in *D. officinale* through transcriptomic analysis [25], while Sun et al. used transcriptomics and metabolomics to reveal the maturation mechanisms of Chinese bayberry during its growth process [26]. Wang et al. applied proteomics to study the pollination response mechanisms of *Dendrobium chrysanthum* [27]. In summary, multi-omics research has been broadly applied to the research of metabolic pathways and other aspects of various plants. However, the biosynthesis pathway of flavonoids in *D. devonianum* flowers remains unclear and requires further investigation.

Flavonoid compounds are the main bioactive components in *D. devonianum* flowers. Investigating the dynamic patterns of flavonoid synthesis and accumulation during different developmental stages, as well as the underlying physiological and molecular mechanisms, is of great significance for ensuring quality and guiding production. Therefore, this study employed transcriptomics and metabolomics to explore the differentially expressed genes associated with flavonoid biosynthesis pathways in *D. devonianum* flowers. It compared the differential metabolites in flowers at various developmental stages and, through a combined analysis of metabolomics and transcriptomics, identified key candidate genes and metabolites involved in the flavonoid biosynthesis pathway. The findings derived from this investigation establish a foundation for subsequent exploration of flavonoid compound accumulation and regulatory mechanisms in *D. devonianum* flowers while creating a theoretical framework to optimize the species’ multifunctional applications.

## 2. Materials and Methods

### 2.1. Plant Material

**Figure 1:**
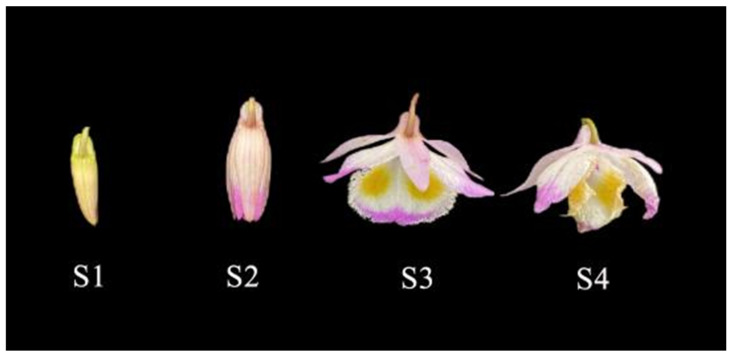
Four developmental periods of *D. devonianum* flowers.

This study utilized *D. devonianum* flowers cultivated at the Dendrobium Germplasm Resource Protection and Research Center in Longling, Baoshan, Yunnan Province, China (98.696° E, 24.593° N). Flowers were collected at four developmental stages: bud stage (S1), initial flowering stage (S2), full bloom stage (S3), and late bloom stage (S4) (Figure 1). Flowers were sampled from plants with consistent growth under the same climatic conditions and management practices. Integrated metabolomic and transcriptomic profiling was performed across all developmental stages, with three biological replicates per stage. Post-collection, samples were immediately flash-frozen in liquid nitrogen and cryopreserved at −80 °C for subsequent analytical procedures.

### 2.2. Metabolite Extraction

Tissues (100 mg) were individually ground with liquid nitrogen, and the homogenate was re-suspended with pre-chilled 80% methanol and 0.1% formic acid by vortexing well. After 5 min incubation on ice, phase separation was achieved by centrifugation at 15,000× *g* (4 °C) for 20 min. The resulting supernatant was standardized to a 53% methanol concentration through dilution with LC-MS grade water. Aliquots were transferred to sterile microcentrifuge tubes for secondary centrifugation under identical parameters (15,000× *g*, 4 °C, 20 min). Processed extracts were ultimately subjected to LC-MS/MS analytical characterization [28].

### 2.3. HPLC-MS/MS Analysis

LC-MS/MS analyses were performed using an ExionLC™ AD system (SCIEX) coupled with a QTRAP^®^ 6500+ mass spectrometer (SCIEX) in Genedenovo (Guangzhou, China). Samples were injected onto an XSelect HSS T3 column (2.1 × 150 mm, 2.5 µm) using positive/negative polarity switching mode with a 20 min linear gradient at a flow rate of 0.4 mL/min. The eluents were eluent A (0.1% formic acid/water) and eluent B (0.1% formic acid/acetonitrile). The solvent gradient was set as follows: 2% B, 2 min; 2–100% B, 15.0 min; 100% B, 17.0 min; 100–2% B, 17.1 min; 2% B, 20min. The QTRAP^®^ 6500+ mass spectrometer was operated in positive polarity mode with a curtain gas of 35 psi, a collision gas of medium, an ion spray voltage of 5500 V, a temperature of 550 °C, an ion source gas of 1: 60, and an ion source gas of 2: 60. The QTRAP^®^ 6500+ mass spectrometer was operated in negative polarity mode with a curtain gas of 35 psi, a collision gas of medium, an ion spray voltage of −4500 V, a temperature of 550 °C, an ion source gas of 1: 60, and an ion source gas of 2: 60.

### 2.4. Metabolites Identification and Quantification

The detection of the experimental samples using MRM (Multiple Reaction Monitoring) was based on an in-house database. The Q3 (the *m*/*z* of the specific product ion generated from the fragmented precursor, detected in the third quadrupole) was used for the metabolite quantification. The Q1 (the mass-to-charge ratio (*m*/*z*) of the intact precursor ion selected in the first quadrupole for fragmentation), Q3, RT (retention time), DP (declustering potential), and CE (collision energy) were used for metabolite identification. The data files generated by HPLC-MS/MS were processed using the SCIEX OS Version 1.4 to integrate and correct the peak. The main parameters were set as follows: minimum peak height, 500; signal/noise ratio, 5; gaussian smooth width, 1. The area of each peak represents the relative content of the corresponding substance.

### 2.5. RNA Sequencing

Total RNA was extracted from *D. devonianum* flowers using the Trizol reagent kit (Invitrogen, Carlsbad, CA, USA) following the manufacturer’s protocol. RNA quality was assessed using an Agilent 2100 Bioanalyzer (Agilent Technologies, Palo Alto, CA, USA) and confirmed by RNase-free agarose gel electrophoresis. After extraction, ribosomal RNA (rRNA) was depleted from prokaryotic mRNA using the Ribo-Zero™ Magnetic Kit (Epicentre, Madison, WI, USA). The enriched mRNA was then fragmented into short pieces using fragmentation buffer, followed by reverse transcription into cDNA using random primers. The second-strand cDNA was synthesized with DNA polymerase I, RNase H, dNTPs, and buffer. cDNA fragments were purified using the QiaQuick PCR Extraction Kit (Qiagen, Venlo, The Netherlands) and ligated to Illumina sequencing adapters. The ligated products were size-selected via agarose gel electrophoresis, PCR-amplified, and sequenced on an Illumina NovaSeq 6000 platform by Gene Denovo Biotechnology Co., Ltd. (Guangzhou, China).

### 2.6. Analysis of RNA Sequencing Data

The expression levels of individual genes were calculated and normalized as RPKM (reads per kilobase of transcript per million mapped reads) [29]. Differential expression analysis between two groups was performed using DESeq2 [30] software (1.20.0), while edgeR [31] software was used for differential expression analysis between two samples (S1 vs. S2, S1 vs. S3, S1 vs. S4, S2 vs. S3, S2 vs. S4, S3 vs. S4). Genes with a false discovery rate (FDR) below 0.05 and an absolute fold change ≥ 2 were considered differentially expressed. GO and KEGG enrichment analyses of the annotated differentially expressed genes were performed using the topGO (2.28.0) and clusterProfiler packages (4.6.1), respectively.

### 2.7. Quantitative Real-Time PCR

Ten genes were selected from the KEGG pathway associated with flavonoid biosynthesis, and the transcriptome data were validated by qRT-PCR. Total RNA was extracted from flowers at different developmental stages using the Trizol method. cDNA was synthesized using a PrimeScript™ RT Kit with gDNA Eraser (Takara, Shiga, Japan) in a reaction volume of 10 μL. The reaction was carried out on a LightCycler 96 real-time PCR system (Roche, Basel, Switzerland). Technical triplicates were performed for each sample. The Actin gene of *D. devonianum* was used as the internal reference for normalization. Relative expression levels were calculated using the 2^−∆∆Ct^ method. The primer sequences were designed using multiPrime [32]. They are shown in Supplementary Table S1.

## 3. Results

### 3.1. Metabolite Profiling of Four Developmental Periods of D. devonianum Flowers

**Figure 2:**
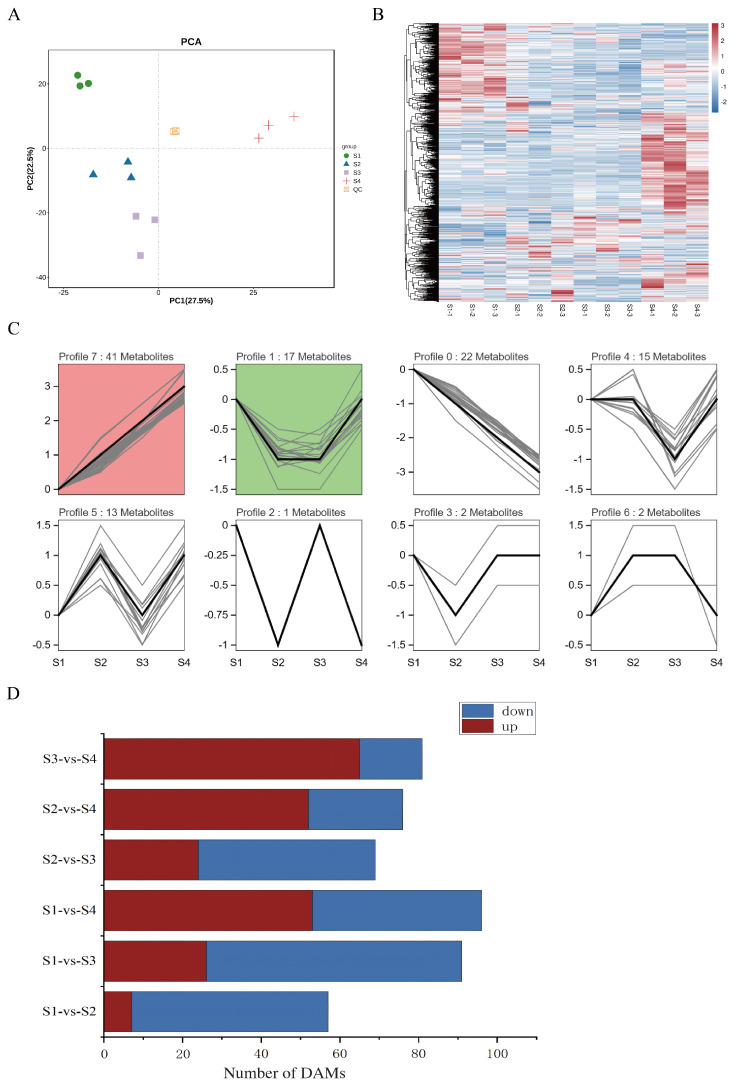
Metabolomic analyses of *D. devonianum* of different growth periods. (**A**) PCA score plots for all samples. (**B**) Clustering heat map of metabolites. (**C**) The K means analysis of flavonoids. modules with color are significantly enriched trend patterns (*p* < 0.05); modules without color are non-significantly enriched trend patterns; modules with similar trends have the same color. (**D**) The number of DAMs in comparison groups.

To elucidate the dynamic changes in metabolite profiles during the development of *D. devonianum* flowers, this study employed a targeted metabolomics approach based on liquid chromatography–tandem mass spectrometry (LC-MS/MS) to analyze samples from four distinct flower developmental stages. Across the four developmental stages of the flowers, a total of 1186 metabolites were identified. The main identified metabolites included flavonoids, amino acids and derivatives, lipids, carbohydrates and their derivatives, as well as organic acids and their derivatives. Notably, flavonoids were the most abundant group, with 213 compounds identified (Table S2). To ensure the reliability of the methodology and data, principal component analysis (PCA) was performed on all compounds detected by LC-MS/MS. The results showed that PC1 and PC2 explained 27.5% and 22.5% of the metabolite variance across all samples, respectively; the four sample groups exhibited clear separation with minimal intra-group variation (Figure 2A). This indicates significant metabolic differences among the developmental stages of *D. devonianum* flowers, confirming the reliability of the data for further analysis. Hierarchical clustering heatmap analysis revealed particularly pronounced changes in metabolites between the S1 and S4 stages (Figure 2B).

To understand the variation trends of flavonoid metabolites during the development of *D. devonianum* flowers, 213 flavonoid metabolites were subjected to time-series clustering analysis using the software Short Time-series Expression Miner (STEM1.3.13). Based on the expression patterns of metabolites, eight clustering modules were identified (Figure 2C). Notably, the flavonoid metabolites in Profile 7 showed a positive correlation with flower development, peaking at the S4 stage. Additionally, to further investigate the metabolic differences among developmental stages, differential metabolites (DAMs) were identified using the criteria of VIP ≥ 1 and T-test P < 0.05. Across six comparison groups (S1 vs. S2, S1 vs. S3, S1 vs. S4, S2 vs. S3, S2 vs. S4, S3 vs. S4), 57, 91, 96, 69, 76, and 81 differential metabolites were detected, respectively. As the flowers matured, the number of significantly upregulated metabolites gradually increased, with the most upregulated metabolites observed in S3 vs. S4 and the fewest in S1 vs. S2. Conversely, S1 vs. S3 had the highest number of downregulated differential metabolites, while S3 vs. S4 had the lowest (Figure 2D). 184 differential metabolites were identified across six comparative groups. Amino acids and their derivatives constituted the most abundant category (47 metabolites), suggesting active synthesis and degradation of amino acids during flowering. This was followed by 29 flavonoids (distributed as 6, 19, 20, 4, 8, and 7 in the S1 vs. S2, S1 vs. S3, S1 vs. S4, S2 vs. S3, S2 vs. S4, and S3 vs. S4 comparisons, respectively), 23 carbohydrates and derivatives, 20 organic acids, 15 phenolic acids, and 15 lipids (Table S3).

**Figure 3:**
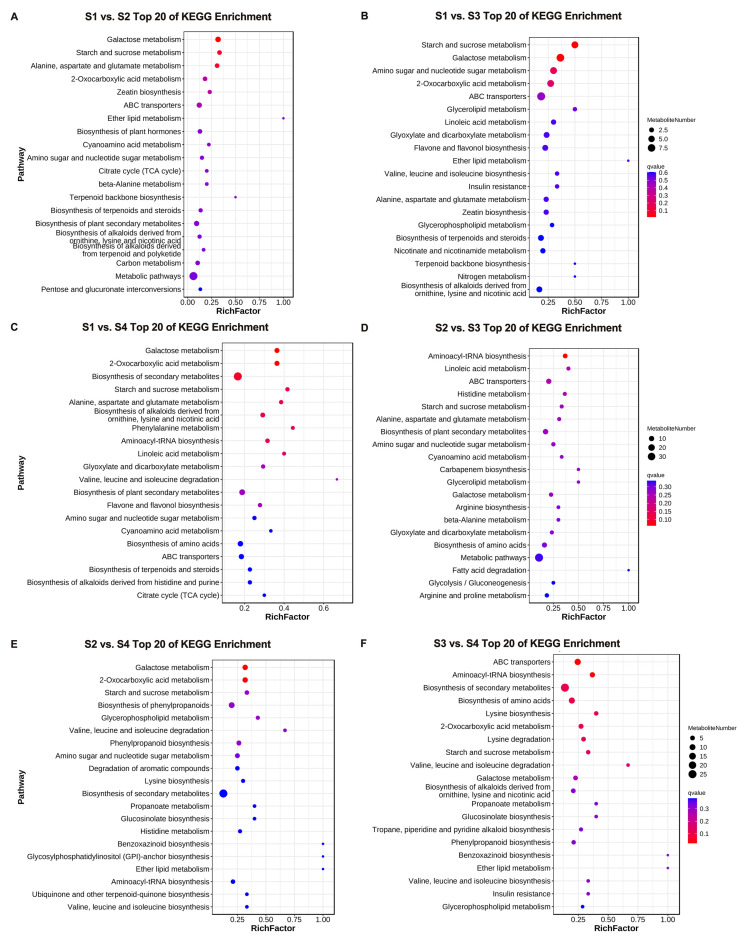
The KEGG pathway analysis of DAMs. (**A**) S1 vs. S2; (**B**) S1 vs. S3; (**C**) S1 vs. S4; (**D**) S2 vs. S3; (**E**) S2 vs. S4; (**F**) S3 vs. S4.

To further elucidate the potential functions of the differential metabolites, KEGG enrichment analysis was conducted on the identified differential metabolites. Figure 3 shows the top 20 enriched pathways in the six comparison groups. These enrichment pathways may be the key to the metabolite changes at different developmental stages of *D. devonianum* flowers. The analysis results showed that differential metabolites were significantly enriched in the pathways of galactose metabolism, starch and sucrose metabolism, amino acid and nucleotide sugar metabolism, 2-oxocarboxylic acid metabolism, and biosynthesis of secondary metabolites in the six comparison groups. In addition, differential metabolites were enriched in the flavone and flavanol biosynthesis pathways in both the S1 vs. S3 and S1 vs. S4 comparison groups.

### 3.2. Transcriptomic Analysis in Four Developmental Stages of D. devonianum Flowers

**Figure 4:**
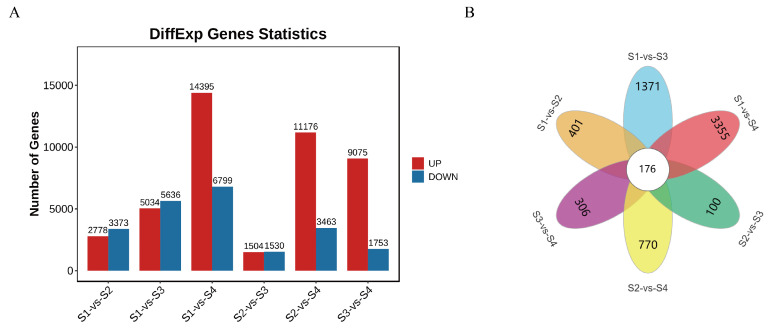
The number of DEGs in comparison groups. (**A**) Statistics of the number of up-regulated (UP) and down-regulated (DOWN) DEGs among the six comparative groups. (**B**) Venn diagram of DEGs in six comparison groups.

To research the dynamic transcriptome changes during the four growth stages of *D. devonianum* flowers and uncover the molecular mechanisms underlying flavonoid metabolite accumulation, RNA-seq analysis was performed on flowers at these developmental stages. The quality control results of the sequencing showed that the proportions of Q20 and Q30 bases ranged from 97.76% to 98.18% and 93.10% to 94.38%, respectively, while the GC content of each sample varied between 44.37% and 45.65% (Table S4), indicating high-quality RNA-seq data. Significant differentially expressed genes (DEGs) were identified in six comparison groups (as mentioned above) based on the criteria of FDR < 0.05 and |log_2_FC| > 1. The results revealed 2778, 5034, 14,395, 1504, 11,176, and 9075 upregulated genes in the S1 vs. S2, S1 vs. S3, S1 vs. S4, S2 vs. S3, S2 vs. S4, and S3 vs. S4 comparison groups, respectively. Similarly, 3373, 5636, 6799, 1530, 3463, and 1753 downregulated genes were identified in these groups (Figure 4A). Among the six comparison groups, 176 DEGs were shared across all groups, while 401, 1731, 3355, 100, 770, and 306 DEGs were uniquely identified in the S1 vs. S2, S1 vs. S3, S1 vs. S4, S2 vs. S3, S2 vs. S4, and S3 vs. S4 groups, respectively (Figure 4B). These findings indicate significant differences in DEGs between the comparison groups.

### 3.3. GO Enrichment and KEGG Pathway Analysis of DEGs

**Figure 5:**
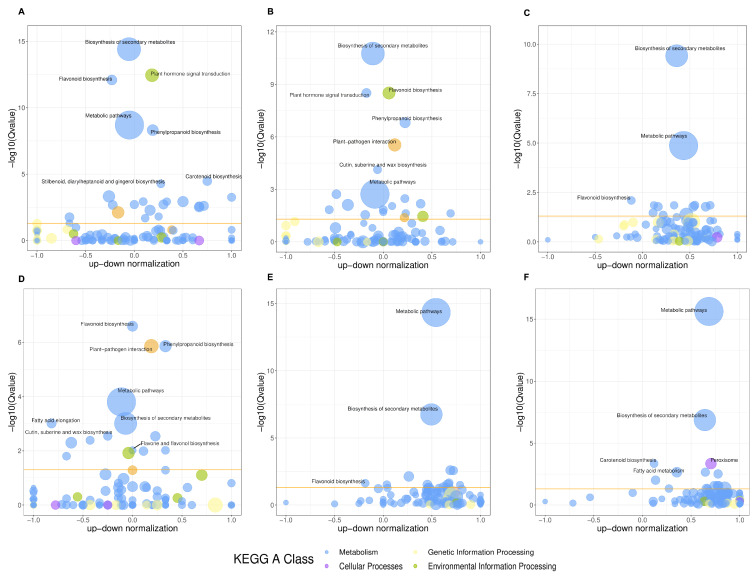
KEGG pathway enrichment bubble diagram. The size of the bubble represents the number of the target DEGs and the color of the bubble represents different KEGG classes. The threshold line in orange is P-value = 0.05. (**A**) S1 vs. S2; (**B**) S1 vs. S3; (**C**) S1 vs. S4; (**D**) S2 vs. S3; (**E**) S2 vs. S4; (**F**) S3 vs. S4.

To functionally describe the DEGs, GO analysis was performed on the differentially expressed genes across the comparison groups. The DEGs were classified into three GO categories: Molecular Function (MF), Cellular Component (CC), and Biological Process (BP). “Cellular process” was the most enriched subcategory in the Biological Process (BP) category, followed by “Metabolic processes”. In the Molecular Function (MF) category, the most enriched subcategories were “Catalytic activity” and “Binding”. For the Cellular Component (CC) category, the DEGs were predominantly enriched in “Cellular anatomical entity” and “Protein-containing complex” (Figure S1). Additionally, KEGG classification and annotation of DEGs were performed to identify major metabolic pathways. The DEGs in the samples were annotated into five KEGG pathway categories, including Metabolism, Genetic Information Processing, Environmental Information Processing, Cellular Processes, and Organismal Systems. A total of 141 pathways were enriched. Among these, Metabolism contained the largest number of annotated pathways and genes, while Organismal Systems had the least. As shown in Figure 5, the top 20 significantly enriched pathways across the six comparison groups were analyzed. Among these, DEGs related to Metabolism accounted for the highest proportion. In all comparison groups, DEGs involved in the “Biosynthesis of secondary metabolites” pathway were significantly enriched. Furthermore, pathways such as “Starch and sucrose metabolism”, “Plant hormone signal transduction”, “Phenylpropanoid biosynthesis”, and “Cutin, suberine, and wax biosynthesis” were also significantly enriched in several comparison groups. Notably, except for the S3 vs. S4 group, the “Flavonoid biosynthesis” pathway was consistently enriched in the top 20 pathways across other comparison groups. Additionally, the “Flavone and flavonol biosynthesis” pathway was significantly enriched in the S2 vs. S4 group. In this study, among the differentially expressed genes (DEGs) identified across all comparison groups, 96 were mapped to flavonoid biosynthesis-related pathways, including “Phenylpropanoid biosynthesis” (KO00940), “Flavonoid biosynthesis” (KO00941), “Anthocyanin biosynthesis” (KO00942), and “Flavone and flavanol biosynthesis” (KO00944). Flavonoids are crucial secondary metabolites in plants; therefore, we conducted a further detailed analysis of the flavonoid biosynthesis pathways.

### 3.4. Metabolic Changes and Transcriptome Regulation Associated with Flavonoid Biosynthesis

**Figure 6:**
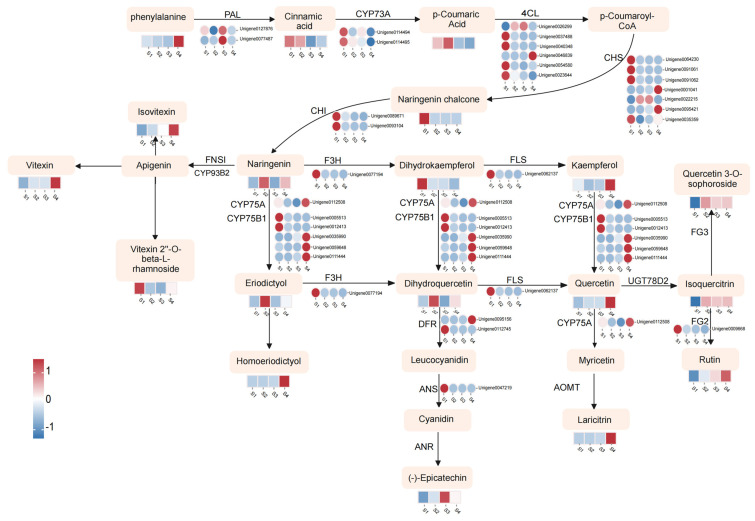
The flavonoids biosynthetic pathway of *D. devonianum* flowers. The heatmap illustrates the expression patterns of DEGs and DAMs during the biosynthesis of flavonoid compounds. Circles represent the expression changes of DEGs, while rectangles denote the expression changes of DAMs. The color scale indicates the relative expression levels and cumulative amounts of DEGs and DAMs, with darker colors signifying higher expression levels. Red indicates upregulation, and blue indicates downregulation. Key enzyme gene abbreviation: *PAL*, phenylalanine ammonia lyase; *CYP73A*, cinnamic acid 4-hydroxylase; *4CL*, 4-coumarate: CoA ligase; *CHS*, chalcone synthase; *CHI*, chalcone isomerase; *F3H*, flavonoid 3-hydroxylase; *FLS*, flavonol synthase; *CYP75A*, flavonoid 3′,5′-hydroxylase; *CYP75B1*, flavonoid 3′-monooxygenase; *DFR*, flavanone 4-reductase; *ANS*, anthocyanidin synthase; *ANR*, Anthocyanidin reductase; *FNS1*, flavone synthase I; *AOMT*, flavonoid O-methyltransferase; *FG2*, flavonol-3-O-glucoside L-rhamnosyltransferase.

Flavonoid compounds are important secondary metabolites in *D. devonianum*. In this study, to visually elucidate the relationship between metabolites and genes in the flavonoid biosynthesis pathway, we integrated differentially accumulated metabolites (DAMs) and differentially expressed genes (DEGs) through KEGG pathway annotation. Within the “Flavonoid biosynthesis” (ko00941) and “Flavone and flavonol biosynthesis” (ko00944) pathways, we identified 31 DEGs, including 2 *PAL*, *CYP73A*, *CHI*, and *DFR* genes; 6 *4CL* genes; 7 *CHS* genes; 5 *CYP75B1* genes; and 1 each of *F3H*, *FLS*, *CYP75A*, *ANS*, and *FG2* genes. Based on these 31 DEGs and KEGG pathways (ko00941 and ko00944), we constructed a comprehensive regulatory network for flavonoid biosynthesis in *D. devonianum* (Figure 6). Significant discrepancies were observed between the expression levels of genes and metabolites during the biosynthesis of flavonoids. In the upstream portion of the pathway, most genes encoding *CYP73A*, *4CL*, *CHS*, and *CHI* were highly expressed in S1 samples, with expression levels decreasing as the flowers developed. *F3H*, a structural gene involved in the hydroxylation of flavonoids to produce dihydrokaempferol, exhibited high expression levels in S1 samples. The expression pattern of the gene encoding *F3H* (*Unigene0077194*) was consistent with the accumulation of dihydrokaempferol. Additionally, kaempferol, quercetin, homoeriodictyol, laricitrin, isovitexin, and vitexin accumulated abundantly in S4 samples. This accumulation may result from the coordinated regulation of numerous genes encoding *FLS*, *CYP75A*, *CYP75B1*, *AOMT*, and *FNSI*. Notably, the expression patterns of *PAL*, *CHI*, *FLS*, *FG2*, *DFR*, and *ANS* genes did not fully align with the trends in metabolite accumulation. This discrepancy may reflect the complexity of gene-to-protein expression processes, with the lack of certain transcription factors potentially being a contributing factor.

### 3.5. Gene Co-Expression Network Analysis

**Figure 7:**
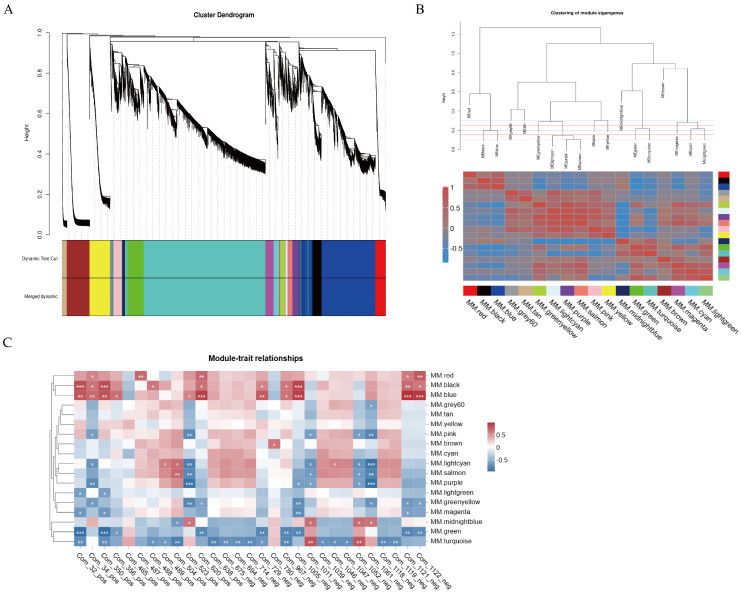
Co-expression network analysis of *D. devonianum* flowers. (**A**) Clustering dendrogram results showing 18 expression modules, each marked with a different color; (**B**) the heatmap of connectivity of eigengenes; (**C**) module–trait associations. Each row represents a module eigengene, and each column represents a trait. “*” indicates the significance of the correlation, the more ‘*’ means the more significant the corre-lation3.6. Quantitative Real-Time PCR Validation.

To find transcripts associated with flavonoid biosynthesis, we conducted a WGCNA analysis using the remaining 16,452 genes from the transcriptome data as the source. First, we constructed a hierarchical clustering tree based on the correlation of gene expression levels, where each branch represented a module, and genes within the same module showed significant co-expression (Figure 7A). Each module was assigned a specific color, and genes that could not be grouped into any other module were classified as gray. The results showed that we identified 18 co-expression modules, with varying numbers of genes. The turquoise module contained the most genes (6347), while the light green module contained the fewest genes (76). To explore the interactions between these gene modules, we performed an adjacency heatmap analysis (Figure 7B), which revealed good correlation and adjacency between the modules. Next, we performed an association analysis between the 29 flavonoid differential metabolites identified by metabolomics and the 18 gene modules generated by WGCNA (Figure 7C). The analysis revealed that five co-expression modules exhibited a strong association with the biosynthesis of the majority of flavonoid metabolites. Specifically, the red, black, and blue modules were positively correlated with most flavonoid metabolites, while the green and turquoise modules showed negative correlations, with other modules showing lower correlations with the traits. in addition. To elucidate the functional roles of these five key modules, we conducted systematic functional classification and annotation of the constituent genes using the KEGG database, with particular emphasis on their potential involvement in metabolic pathways. The results demonstrated that genes within the black module exhibited significant enrichment in metabolic pathways related to phenylpropanoid biosynthesis, biosynthesis of secondary metabolites, flavonoid biosynthesis, and flavone and flavonol biosynthesis. In the blue, red, green, and turquoise modules, genes were significantly enriched in metabolic pathways such as proteasome, photosynthesis, spliceosome, and DNA replication (Figure S2). These findings further suggest that the black module is closely associated with flavonoid metabolism.

To validate the reliability and accuracy of the transcriptome data, we randomly selected 10 DEGs involved in flavonoid biosynthesis and conducted qPCR analysis on RNA samples from four different developmental stages. The results showed that the expression trends of the genes in qRT-PCR were generally consistent with those observed in the transcriptome data, confirming the reliability of the transcriptome data (Figure S3).

## 4. Discussion

The flowers of *D. devonianum* contain a diverse array of secondary metabolites. In this study, HPLC-MS/MS was employed to analyze the flowers at four different developmental stages. A total of 1186 compounds were detected across the four sample groups, with flavonoids being the most abundant, comprising 213 different types. Flavonoids constitute a functionally vital class of specialized metabolites in plants, known for their anticancer, antitumor, and antioxidant properties [33]. They are widely distributed in medicinal plants and have been isolated from species such as *Ginkgo biloba* leaves [34], *Radix Scutellariae* [35], and *Carthamus tinctorius* [36]. Metabolomic analysis revealed that flavonoids predominated among differentially accumulated metabolites (DAMs), with 29 distinct flavonoid compounds identified. These include various quercetin derivatives such as quercetin-3,4′-O-di-β-glucopyranoside, quercetin 5-O-hexoside, quercetin-O-glucoside, isoquercitrin, quercetin-3′-O-glucoside, rutin, and isotrifoliin. Quercetin and its glycoside derivatives are typical flavonoids known for their antitumor, antiviral, antidiabetic, and cardiovascular protective effects [37]. Cluster analysis of flavonoid metabolite trends revealed that the expression trends of 41 flavonoid metabolites were positively correlated with the development of *D. devonianum* flowers and peaked at the S4 period. In addition, the analyses showed that there were significant differences in metabolite expression in *D. devonianum* flowers at different developmental periods. In contrast, metabolite expression was higher in S1 and S4, suggesting that harvesting *D. devonianum* flowers at S1 or S4 could obtain a greater amount of compound efficacy.

Transcriptome analysis revealed that 96 DEGs were mapped to flavonoid biosynthesis-related pathways, including “Phenylpropanoid biosynthesis”, “Flavonoid biosynthesis”, “Anthocyanin biosynthesis”, and “Flavone and flavonol biosynthesis”. Most DEGs involved in flavonoid-related synthetic pathways were significantly upregulated in the S1 and S4 samples, which is not consistent with the expression trend of flavonoid differential metabolites and which reflects the complexity between flavonoid biosynthesis and gene regulation. The synthesis of flavonoid compounds in plants is relatively conserved, and their biosynthesis occurs through multiple pathways and is regulated by several structural genes [38]. The majority of genes involved in this biosynthetic pathway have been cloned and functionally validated in several model plant species [39]. Currently, genes associated with the flavonoid synthesis pathway in *Dendrobium officinale* have been identified, including *CHS*, *DFR*, *FLS*, and *F3H* genes [40]. Using transcriptomic data, we identified 31 DEGs involved in the “Flavonoid biosynthesis” pathway, including *PAL*, *CYP73A*, *CHI*, *DFR*, *4CL*, *CHS*, *CYP75B1*, *F3H*, *FLS*, *CYP75A*, *ANS*, and *FG2*. Among these, most *CYP73A*, *4CL*, *CHS*, *CHI*, *F3H*, and *FLS* genes exhibited higher expression levels in the S1 stage. The co-expression of these genes may contribute to flavonoid compound biosynthesis and could explain the increased accumulation of flavonoids in the flowers of *D. devonianum*.

The study of flavonoid biosynthesis pathways highlights that the biosynthesis of phenylpropanoids is a complex and vital process. Previous research indicates that the phenylpropanoid pathway comprises the initial three steps of flavonoid biosynthesis [41]. In this pathway, phenylalanine is converted into p-coumaroylCoA through the catalytic actions of phenylalanine ammonia-lyase (*PAL*) and 4-coumarate-CoA ligase (*4CL*) [42]. Subsequently, p-coumaroyl-CoA is transformed into flavonoids through the sequential actions of chalcone synthase (*CHS*), chalcone isomerase (*CHI*), flavonol synthase (*FLS*), dihydroflavonol reductase (*DFR*), and anthocyanidin reductase (*ANR*) [43,44,45]. We investigated the changes in the transcriptome and metabolome of *D. devonianum* flowers at different developmental stages and integrated the results of DAMs and DEGs in the flavonoid biosynthesis pathway. The results indicate that genes and metabolites associated with flavonoid compounds undergo significant changes at different developmental stages. In the upstream steps of flavonoid biosynthesis, phenylalanine is first converted into p-CoumaroylCoA under the catalysis of *PAL* and *4CL*. Subsequently, 4-coumaroyl-CoA is transformed into naringenin, a key intermediate in flavonoid biosynthesis, through the catalytic actions of *CHS* and *CHI*. Naringenin then branches into three pathways: one leading to the production of flavonoids such as vitexin, isovitexin, apigenin, and vitexin-2-O-rhamnoside; the second leading to flavonols such as dihydrokaempferol, kaempferol, and quercetin; and the third branching into the production of eriodictyol and homoeriodictyol. These findings are similar to previous studies [46,47]. It has been reported that naringenin exhibits a wide range of biological activities, including antioxidant, hepatoprotective, anti-inflammatory, and anticancer effects [48]. Therefore, further exploration of the pharmacological potential of flavonoid compounds in *D. devonianum* flowers is warranted.

Transcriptome and metabolome analyses indicate that the gene encoding *CHS* is significantly associated with downstream flavonoid compounds. *CHS* shows higher expression in the S1 stage, and its catalytic product, naringenin chalcone, also exhibits higher expression at this stage. This is consistent with the expression patterns of downstream metabolites, providing a foundation and precursor for the accumulation of flavonoids. In fact, *CHS* plays a critical role in flavonoid biosynthesis, acting as a key enzyme in the production of important flavonoids. Studies have shown that *CHS* serves as an enzyme in the biosynthesis of naringenin [49], controlling the first step in flavonoid biosynthesis by catalyzing the conversion of three molecules of malonyl-CoA and one molecule of p-coumaroyl-CoA into naringenin chalcone. This is followed by rapid conversion to naringenin via chalcone isomerase (*CHI*) and further flavonoid synthesis through downstream enzymes [50,51]. Therefore, we hypothesize that the high expression of upstream genes and metabolites might promote the accumulation of downstream flavonoids. Furthermore, it is worth noting that four genes—*F3H*, *FLS*, *CYP75A*, and *CYP75B1*—show significant changes across different growth years and catalyze multiple processes. For example, F3H catalyzes the conversion of naringenin to dihydrokaempferol. Flavanone-3-hydroxylase (*F3H*) is one of the key enzymes in the flavonoid biosynthesis pathway, catalyzing the hydroxylation of the C-ring at position 3 of naringenin to form dihydrokaempferol [52]. Studies have shown that the expression of the *F3H* gene is closely related to anthocyanin content, thereby influencing plant pigmentation. Overexpression of the *F3H* gene in *Torenia fournieri* leads to changes in flower color [53], and transferring the *F3H* gene from *Rhododendron hybridum* into *Arabidopsis thaliana* increases anthocyanin accumulation in transgenic plants [54]. Lack of F3H enzyme activity results in white flowers in species such as *Dahlia pinnata*, *Verbena officinalis*, and *Zinnia elegans* [55]. In *Arabidopsis* mutant tt6-5, mutation in the AtF3H gene leads to an increase in naringenin content, a decrease in dihydrokaempferol levels, and a significant reduction in total flavonoids and anthocyanins [56]. These findings suggest that *F3H* is a regulatory node in the flavonoid biosynthesis pathway in *D. devonianum* flowers, playing an important role in the secondary metabolic process. *FLS* catalyzes the conversion of dihydrokaempferol to kaempferol and dihydroquercetin to quercetin. Research indicates that *FLS* is a key regulatory enzyme in the flavonoid biosynthesis pathway, influencing the production of secondary metabolites such as kaempferol and quercetin. It catalyzes flavonol synthesis and competes with the anthocyanin biosynthesis enzyme *DFR* for common substrates [57,58]. The *CYP75* gene family consists of two subfamilies, *CYP75A* (flavonoid 3′,5′-hydroxylase, *F3′5′H*) and *CYP75B* (flavonoid 3′-hydroxylase, *F3′H*), which catalyze the hydroxylation of the B-ring in flavonoids. This is a critical biosynthetic process in the synthesis of cyanidin and delphinidin, the precursors of blue and red anthocyanins [59]. *CYP75B1* catalyzes the 3′-hydroxylation of the flavonoid B-ring, converting it to a 3′,4′-hydroxy state, transforming naringenin into eriodictyol and dihydrokaempferol into dihydroquercetin. F3H, FLS, *CYP75B1*, and *CYP75A* genes show relatively high expression in both the S1 and S4 stages. Therefore, we hypothesize that the high expression of *CHS*, *F3H*, *FLS*, *CYP75B1*, and *CYP75A* may play a key role in the biosynthesis of flavonoid compounds in *D. devonianum* flowers and contribute significantly to the accumulation of flavonoids. Further validation of their functions is needed.

## 5. Conclusions

In summary, this study provides a comprehensive analysis of the transcriptome and metabolome of *D. devonianum* flowers, revealing the regulatory network involved in the biosynthesis of flavonoid compounds and identifying key regulatory genes. This research offers valuable insights into the formation mechanisms of active compounds in *D. devonianum* flowers, serving as an important reference for further studies. Therefore, the findings of this study lay a theoretical foundation for improving the quality of *D. devonianum* flowers and for the development of related products.

## Data Availability

The original contributions presented in the study are included in the article/Supplementary Material, further inquiries can be directed to the corresponding author(s).

## Supplementary Materials

The following supporting information can be downloaded at: https://www.mdpi.com/article/10.3390/genes16030264/s1, Table S1: Genes IDs and primers used in the quantitative real-time PCR (qRT-PCR) experiments; Table S2. All metabolites in the flower of *D. devonianum*; Table S3. Different metabolites in the flower of *D. devonianum*; Table S4. Statistical analysis of transcriptome sequencing quality in four periods of *D. devonianum* flowers; Figure S1. GO enrichment of DEGs; Figure S2. KEGG enrichment analysis of five modules; Figure S3. Quantitative real time-PCR validation.
