# Supplementary material for: Metabolomic and Transcriptomic Analyses of Flavonoid Biosynthesis in Dendrobium devonianum Flowers
Source: Genes (Basel). 2025 Feb 24;16(3):264. doi: 10.3390/genes16030264 (PMC11942320; doi:10.3390/genes16030264)
Supplement: Supplementary file 1 [file genes-16-00264-s001.zip › genes-3472812-supplementary.pdf]

Supplemental files

Table S1 Genes IDs and primers used in the quantitative real-time PCR (qRT-PCR) experiments.

Primer_ID	Forward PCR Primer (5′-3′)	Reverse PCR Primer (5′-3′)
Unigene0001041 (*CHS*)	CATGAGAGGGCCAGTTTCGT	CAAATGGGGAGAACGCCTCT
Unigene0089671 (*CHI*)	CCGTCATCGGAGTGTACCTG	GTTGACCCGTTAATGGCAGC
Unigene0062137 (*FLS*)	GCCCACACTGATTTGTCTGC	CACAAAAACCGGCCATGACA
Unigene0117341 (*4CL2*)	TGAGGTGCCTGTAGCCTTTG	TTCCCCGATGGTGATTTGGG

Table S2. All metabolites in the flower of D. *devonianum.*

Class	number
Flavonoids	213
Amino Acid And Derivatives	188
Lipids	109
Carbohydrates And Its Derivatives	106
Organic Acid And Its Derivatives	106
Nucleotide And Its Derivates	74
Organoheterocyclic compounds	68
Phenolic acids	61
Phenylpropanoids and polyketides	50
Terpenoids	50
Phenols And Its Derivatives	46
Alkaloids and derivatives	28
Amines	27
Vitamins	21
Phytohormones	19
Alcohols and polyols	9
Organooxygen compounds	4
Benzene and substituted derivatives	3
Polyamine	3
Organosulfur compounds	1

Table S3. Different metabolites in the flower of D. *devonianum.*

Class	number
Amino Acid And Derivatives	47
Flavonoids	29
Carbohydrates And Its Derivatives	23
Organic Acid And Its Derivatives	20
Phenolic acids	15
Lipids	15
Nucleotide And Its Derivates	9
Organoheterocyclic compounds	8
Phytohormones	4
Phenylpropanoids and polyketides	4
Vitamins	3
Alkaloids and derivatives	3
Amines	2
Phenols And Its Derivatives	1
Organooxygen compounds	1

Table S4. Statistical analysis of transcriptome sequencing quality in four periods of **D. *devonianum*** flowers.

**Sample**	**Raw reads**	**Clean reads (%)**	**Q20(%)**	**Q30(%)**	**GC(%)**
S1-1	5847975300	5817433185(99.48%)	98.06%	94.09%	45.21%
S1-2	7302054300	7267894323(99.53%)	98.15%	94.29%	45.02%
S1-3	7081612500	7047394375(99.52%)	98.18%	94.38%	45.21%
S2-1	6310809300	6279454083(99.50%)	98.08%	93.73%	44.37%
S2-2	7176426300	7127007202(99.31%)	97.93%	93.54%	45.65%
S2-3	5987607300	5945076387(99.29%)	98.10%	94.23%	45.63%
S3-1	7234611000	7188920316(99.37%)	97.87%	93.37%	45.07%
S3-2	7140007200	7106878683(99.54%)	97.96%	93.49%	44.91%
S3-3	7031886000	6983051858(99.31%)	97.76%	93.10%	44.57%
S4-1	7184385300	7138107088(99.36%)	97.93%	93.55%	45.55%
S4-2	7305805200	7252782283(99.27%)	98.01%	93.99%	45.30%
S4-3	7191863700	7148150658(99.39%)	98.06%	94.15%	45.09%

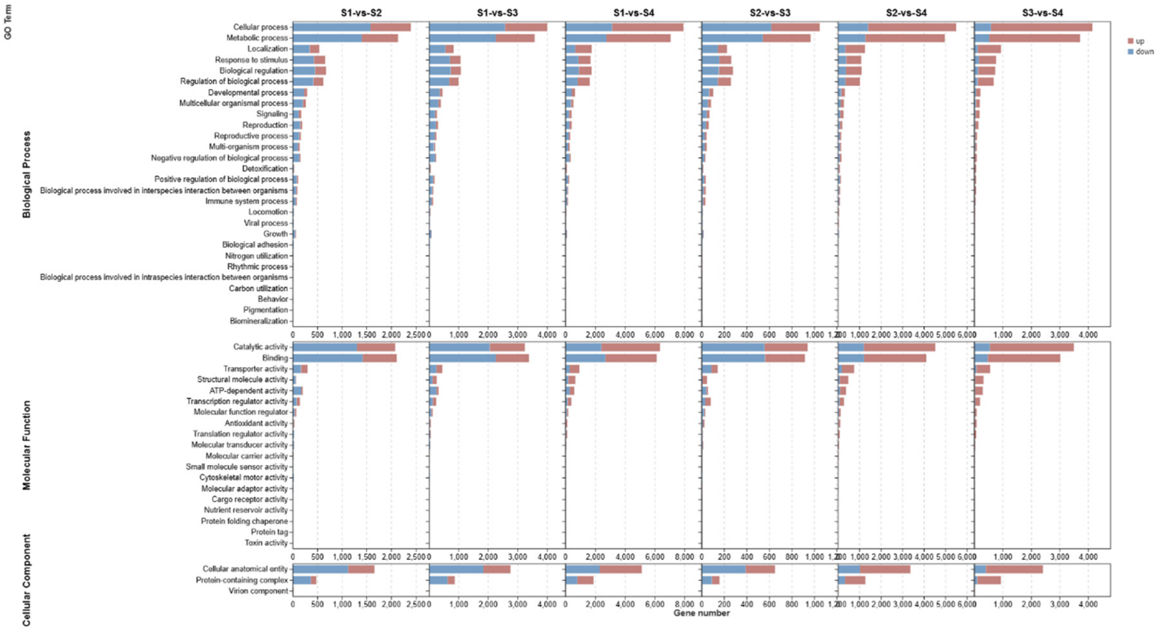

Figure S1. GO enrichment of DEGs.

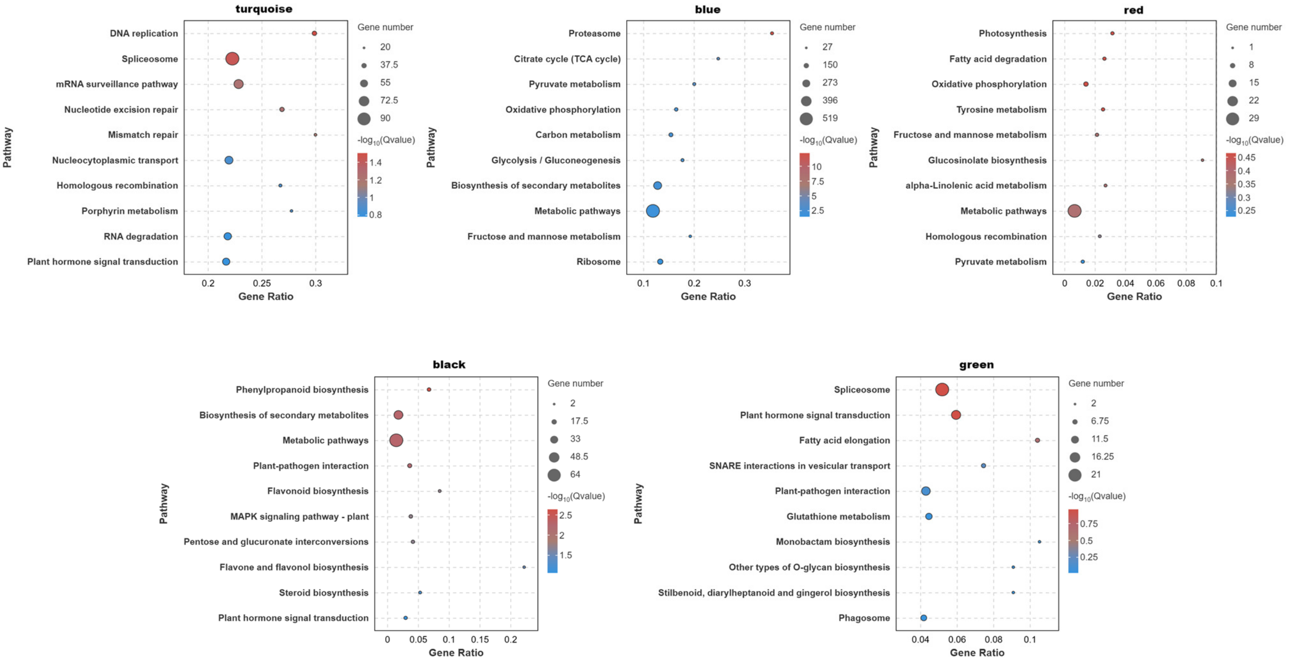

Figure S2. KEGG enrichment analysis of five modules

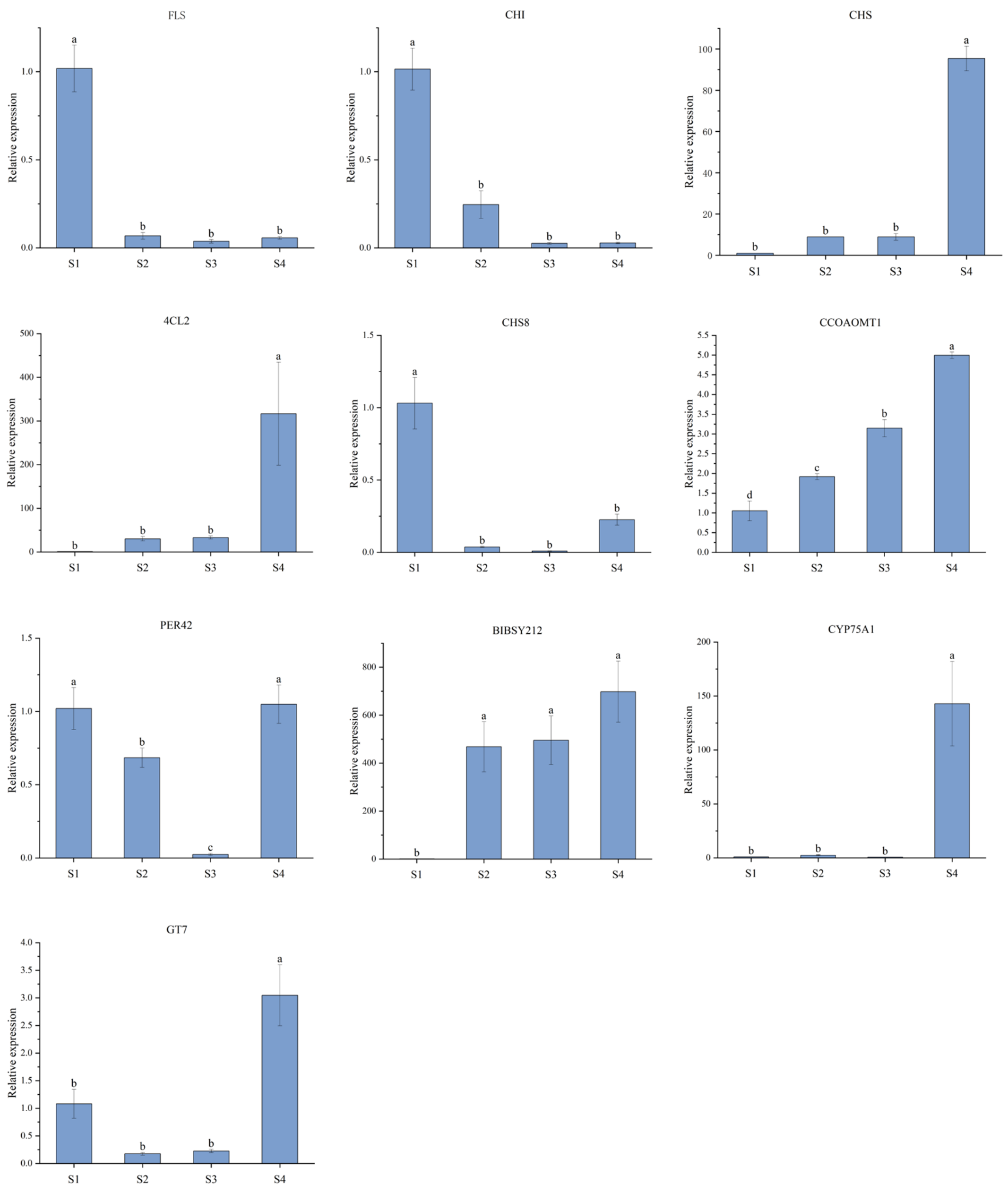

Figure S3. Quantitative real time-PCR validation. Values are mean ± SD (n = 3 independent measurements); different lowercase letters (a, b, c, d) indicate significant differences, $p < 0.05$.
